# Supplementary material for: Coinfection with chytrid genotypes drives divergent infection dynamics reflecting regional distribution patterns
Source: Commun Biol. 2023 Sep 14;6:941. doi: 10.1038/s42003-023-05314-y (PMC10502024; doi:10.1038/s42003-023-05314-y)
Supplement: Supplementary file 2 — Supplementary Material [file 42003_2023_5314_MOESM2_ESM.pdf]

Supplementary Information Appendix

Coinfection with chytrid genotypes drives divergent infection dynamics reflecting regional distribution patterns

Tamilie Carvalho[*,°], Daniel Medina[°], Luisa P. Ribeiro, David Rodriguez, Thomas S. Jenkinson, C. Guilherme Becker, Luís Felipe Toledo[°], Jessica Hite

[*]To whom correspondence should be addressed: tamilie@umich.edu

[°]These authors contributed equally.

Supplementary Result section:

Analysis assessing the effect of the variation in inoculum dose on metrics of genotype competitiveness, overall virulence and transmission potential.

Initial pathogen dose contributed little to host life span, competitiveness, or transmission potential (all terms $p > 0.05$). The initial pathogen dose did not significantly influence early pathogen establishment across all genotypes (gamma glm, $F_{1,146} = 66.86$, $p = 0.716$; Fig. 1A) nor when accounting for genotype-specific differences ($F_{9,138} = 3.86$, $p = 0.722$). Our results suggest that for the focal genotypes used here (P1 and P2), each additional increase in initial pathogen dose increased pathogen establishment by approximately 2.445^{-07} $\text{zoospores}^{-\text{mL}}$. Across all pathogen genotypes, the initial exposure also dose did not significantly influence host life span (gamma glm, mean day of death, $F_{1,55} = 1{,}742$, $p = 0.318$; Fig. 1B) nor when accounting for genotype-specific differences ($F_{4,52} = 560.2$, $p = 0.340$). This result suggests that an additional increase in initial pathogen dose increased host mortality by -1.831^{-07} $\text{days}^{-\text{individual}}$. Lastly, across all genotypes, the initial pathogen dose did not significantly influence transmission potential (total pathogen load; gamma glm, $F_{1,148} = 838$, $p = 0.318$; Fig. 1C), nor were there genotype-specific differences (gamma glm, $F_{9,140} = 4{,}845$, $p = 0.913$).

Supplementary Table 1. Overview of coinfection experiments illustrating typical experimental designs of either holding pathogen dose constant or increasing pathogen dose (and virulence) during coinfection assays.

Number	Authors	Year	Journal	Same pathogen species?	Coinfection dose constant?	DOI
1	Ramsay and Rohr	2022	Journal of Applied Ecology	Yes	Yes, of the focal strain, but examined a range of doses for 'coinfecting' strain	doi.org/10.1111/1365-2664.14332
2	de Roode et al.	2005	The American Naturalist	Yes	No	doi.org/10.1086/491659
3	Ben-Ami et al.	2008	Evolution	Yes	No	doi.org/10.1111/j.1558-5646.2008.00391.x
4	Ge et al.	2012	Virology	No	Yes	doi.org/10.1186/1743-422X-9-128
5	Niczyporuk et al.	2014	Pol J Vet Sci	Yes	Yes, of the focal strain but examined a range of doses for 'coinfecting' strain	doi.org/10.2478/pjvs-2014-0001
6	Bell et al.	2006	Evolution	Yes	No	doi.org/10.1111/j.0014-3820.2006.tb01215.x
7	Blaustein et al.	2018	Diversity	Yes	Variable	doi.org/10.3390/d10030081
8	Jenkinson et al.	2018	Proc B	Yes	No	doi.org/10.6084/m9.figshare.c.4320824.v1
9	Thompson et. al.	2017	Scientific Reports	Yes	Yes	doi.org/10.1038/s41598-017-00835-z
10	Gipson et. al.	2019	Evolution	Yes	Yes	doi.org/10.1111/evo.13760
11	Ben-Ami et al.	2011	J. Evolutionary Biology	No	No	doi.org/10.1111/j.1420-9101.2011.02264.x
12	Seppalla et al.	2012	The American Naturalist	Both	Both	doi.org/10.1086/666985
13	Taylor et al.	1997	Proc B	Yes	No	doi.org/10.1098/rspb.1997.0128
14	Clay et al.	2019	The American Naturalist	No	No	doi.org/10.1086/701126
15	Clay et al.	2020	Proc B	No	No	doi.org/10.1098/rspb.2020.0046
16	deRoode et al.	2005	PNAS	Yes	No	doi.org/10.1073/pnas.05000781
17	Karvonen et al.	2011	Proc B	Yes	Yes	doi.org/10.1098/rspb.2011.0879
18	Klemme et al.	2016	Journal of Animal Ecology	Yes	Yes	doi.org/10.1111/1365-2656.12472
19	Susi et al.	2015	Nature Communications	Yes	Yes	doi.org/10.1038/ncomms6975
20	Kinnula et al.	2017	BMC Evol Biol	Yes	Yes	doi.org/10.1186/s12862-017-0922-2
21	Longo et al.	2019	Biological Invasions	No	Yes	doi.org/10.1007/s10530-019-01973-3
22	Friday at a.	2020	J Exp Zool A Ecol Integr Physiol	No	Yes	doi.org/10.1002/jez.2427

Supplementary Table 2. Treatment groups of the infection assay with five different genotypes of the fungus *Batrachochytrium dendrobatidis* (Bd), which included single-genotype and mixed genotype exposures with either two or three genotypes in all possible combinations, and a control group. Designations (panzootic 'P', enzootic 'E' and hybrid 'H'), number of hosts per treatment, and chytrid lineages or genotype.

Treatment Groups	n	Lineages or genotype
P1	16	Bd-GPL
P2	16	Bd-GPL
E1	16	Bd-Asia-2/Brazil
E2	16	Bd-Asia-2/Brazil
H	16	Hybrid
P1 + E1	15	Bd-GPL + Bd-Asia-2/Brazil
P1 + E2	15	Bd-GPL + Bd-Asia-2/Brazil
P1 + H	14	Bd-GPL + Hybrid
P2 + E1	14	Bd-GPL + Bd-Asia-2/Brazil
P2 + E2	16	Bd-GPL + Bd-Asia-2/Brazil
P2 + H	15	Bd-GPL + Hybrid
P1 + E1 + H	16	Bd-GPL + Bd-Asia-2/Brazil + Hybrid
P1 + E2 + H	16	Bd-GPL + Bd-Asia-2/Brazil + Hybrid
P2 + E1 + H	16	Bd-GPL + Bd-Asia-2/Brazil + Hybrid
P2 + E2 + H	16	Bd-GPL + Bd-Asia-2/Brazil + Hybrid
Control	32	Control

Supplementary Table 3. Applied Biosystems assay information for BdSC9_621917_AC (Assay ID AHBKG6X) at 40X concentration.

Primer/Probe	Sequence	Concentration (μM)	Reporter	Quencher
Forward primer	GAGCTGGCCTTTCTCTTGAGA	36	-	-
Reverse primer	CGTAGAATAAGAAGACATTGCACTTGGT	36	-	-
Reporter 1	AGATCAAAATGGTCACTCAT	8	VIC	NFQ
Reporter 2	CAAAATGGGCACTCAT	8	FAM	NFQ

Supplementary Table 4. Standard curve parameters for assay Bdmt_26360_AC, when 1.34E-5 ng/μl, 1.34E-4 ng/μl, 0.00134 ng/μl, 0.0134 ng/μl, 0.134 ng/μl of DNA were used as input for strains BAF01 (Bd-Asia-2/Brazil), NAF01 (Bd-GPL), and CLFT024-02 (hybrid). Reactions were run in triplicate.

Reporter	Allele	Target genome	Slope	Y-intercept	R^2	Efficiency (%)	Std. dev.	Std. error
FAM	A	BAF 01	-3.4276	20.5516	0.9984	95.7714	0.0330	0.0506
VIC	G	NAF 01	-3.2988	21.4904	0.9930	100.9743	0.1757	0.0765
VIC	G	CLFT 024-02	-3.4318	20.2440	0.9992	95.6105	0.0192	0.0377

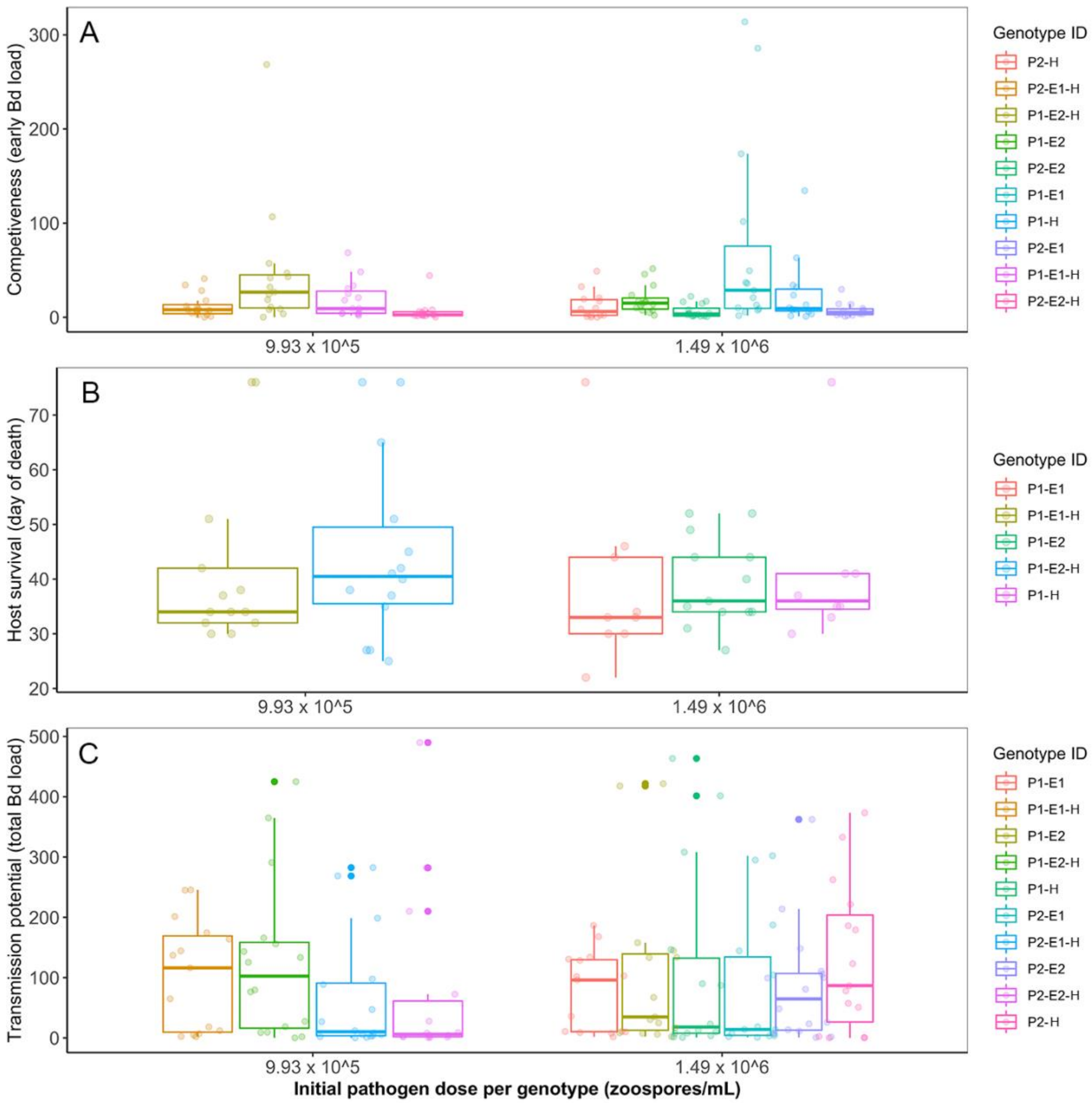

Supplementary Figure 1. The initial pathogen dose did not significantly influence competitiveness (early pathogen establishment (A), host survival (B) and transmission potential (C). Exposure treatments associated with pathogen doses $9.93x10^5$ and $1.49x10^6$ represent triple and double infections, respectively. Data represent the median, upper and lower quartile, and maximum and minimum values. Infection loads are represented by Bd concentration in pg/µl.

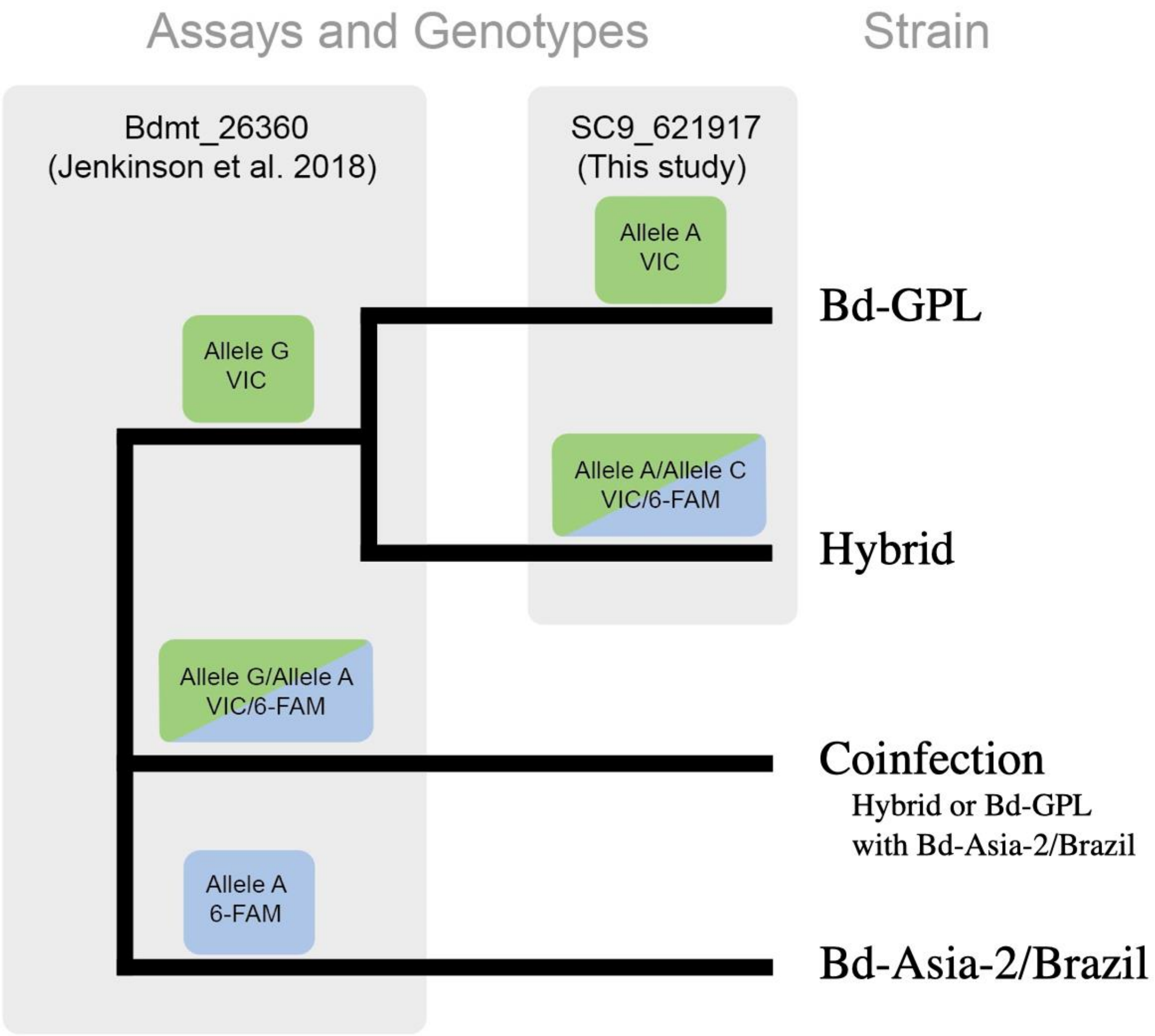

Supplementary Figure 2. Diagram of diagnostic genotyping assays used in this study.

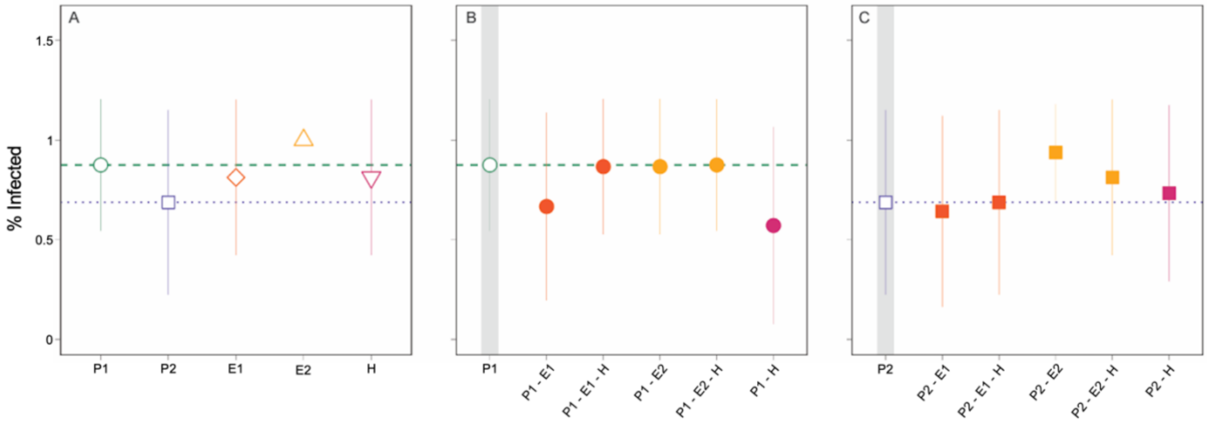

Supplementary Figure 3. The presence of multiple genotypes did not affect the proportion of hosts infected (Binomial GLM, all p-values > 0.05). Gray bars highlight single-genotype infections. These results serve as a quality control metric and clarify that our infection methodology was consistent across treatments.
