# Supplementary material for: Coinfection with chytrid genotypes drives divergent infection dynamics reflecting regional distribution patterns
Source: Commun Biol. 2023 Sep 14;6:941. doi: 10.1038/s42003-023-05314-y (PMC10502024; doi:10.1038/s42003-023-05314-y)
Supplement: Supplementary file 3 — Description of Additional Supplementary Files [file 42003_2023_5314_MOESM3_ESM.pdf]

Description of Additional Supplementary Files

File name: Supplementary Data 1
Description: Sequence data in fasta format used to explore the phylogenetic relationships between the *Batrachochytrium dendrobatidis* (Bd) isolates used in this study.

File name: Supplementary Data 2
Description: *Batrachochytrium dendrobatidis* (Bd) genotypes isolated from amphibians across Brazilian Atlantic Forest. This table includes isolate name or museum record of the host, host species, sampled host life-stage (tadpole or adult) and breeding habitat, collector name, isolation year, chytrid lineage or genotype, municipality, Brazilian state, latitude (Lat), longitude (Lon), and reference.
