# Supplementary material for: Coinfection with chytrid genotypes drives divergent infection dynamics reflecting regional distribution patterns
Source: Commun Biol. 2023 Sep 14;6:941. doi: 10.1038/s42003-023-05314-y (PMC10502024; doi:10.1038/s42003-023-05314-y)
Supplement: Supplementary file 6 — Reporting Summary [file 42003_2023_5314_MOESM6_ESM.pdf]

nature portfolio

Corresponding author(s): COMMSBIO-22-2981D

Last updated by author(s): Aug 25, 2023

Reporting Summary

Nature Portfolio wishes to improve the reproducibility of the work that we publish. This form provides structure for consistency and transparency in reporting. For further information on Nature Portfolio policies, see our Editorial Policies and the Editorial Policy Checklist.

Statistics

For all statistical analyses, confirm that the following items are present in the figure legend, table legend, main text, or Methods section.

n/a	Confirmed	
☐	☒	The exact sample size (n) for each experimental group/condition, given as a discrete number and unit of measurement
☐	☒	A statement on whether measurements were taken from distinct samples or whether the same sample was measured repeatedly
☐	☒	The statistical test(s) used AND whether they are one- or two-sided<br>*Only common tests should be described solely by name; describe more complex techniques in the Methods section.*
☐	☒	A description of all covariates tested
☐	☒	A description of any assumptions or corrections, such as tests of normality and adjustment for multiple comparisons
☐	☒	A full description of the statistical parameters including central tendency (e.g. means) or other basic estimates (e.g. regression coefficient) AND variation (e.g. standard deviation) or associated estimates of uncertainty (e.g. confidence intervals)
☐	☒	For null hypothesis testing, the test statistic (e.g. F, t, r) with confidence intervals, effect sizes, degrees of freedom and P value noted<br>*Give P values as exact values whenever suitable.*
☒	☐	For Bayesian analysis, information on the choice of priors and Markov chain Monte Carlo settings
☐	☒	For hierarchical and complex designs, identification of the appropriate level for tests and full reporting of outcomes
☐	☒	Estimates of effect sizes (e.g. Cohen's d, Pearson's r), indicating how they were calculated

Our web collection on statistics for biologists contains articles on many of the points above.

Software and code

Policy information about availability of computer code

Data collection	Not applicable
Data analysis	Not applicable

For manuscripts utilizing custom algorithms or software that are central to the research but not yet described in published literature, software must be made available to editors and reviewers. We strongly encourage code deposition in a community repository (e.g. GitHub). See the Nature Portfolio guidelines for submitting code & software for further information.

Data

Policy information about availability of data

All manuscripts must include a data availability statement. This statement should provide the following information, where applicable:

- Accession codes, unique identifiers, or web links for publicly available datasets
- A description of any restrictions on data availability
- For clinical datasets or third party data, please ensure that the statement adheres to our policy

Data and R code used to conduct the formal analyses of this manuscript are available at Dryad (https://doi.org/10.5061/dryad.cc2fqz693)

Human research participants

Policy information about studies involving human research participants and Sex and Gender in Research.

Reporting on sex and gender	Not applicable
Population characteristics	Not applicable
Recruitment	Not applicable
Ethics oversight	Not applicable

Note that full information on the approval of the study protocol must also be provided in the manuscript.

Field-specific reporting

Please select the one below that is the best fit for your research. If you are not sure, read the appropriate sections before making your selection.

☐ Life sciences ☐ Behavioural & social sciences ☒ Ecological, evolutionary & environmental sciences

For a reference copy of the document with all sections, see nature.com/documents/nr-reporting-summary-flat.pdf

Ecological, evolutionary & environmental sciences study design

All studies must disclose on these points even when the disclosure is negative.

Study description	We conducted a factorial experiment with 17 different pathogen exposure treatments and one control. The experiment included 297 experimental units (n=14-16 frogs per treatment; control n=32).
Research sample	Adult females and males of the frog species Eleutherodactylus johnstonei (Anura; Eleutherodactylidae) were all collected from the same population in the city of São Paulo. This population was chosen because it is an invasive population and free of the pathogenic chytrid fungus.
Sampling strategy	We used a non invasive sampling techinique (skin swab) to detected and quantifying the pathogen load. Our sample size was based on previous work published by our research group.
Data collection	Tamilie Carvalho (TC) collected all swabs during the experiment. To swab the frogs, the ventral part of the body and limbs were rubbed 5 times each with the tip of a cotton swab.
Timing and spatial scale	TC collected swabs three times for each animal throughout the experiment that started on 04-01-2018 and ended 76 days later. The first swab was collected before the exposure experiment to test whether the animals were negative for the chytrid fungus. To quantify Bd infection following exposure, TC collected skin swabs on day 21 post exposure (to access the pathogen load at the early stage of infection) and after death or at the end of the experiment (day 76, to access the pathogen load in the final stage of infection).
Data exclusions	We did not exclude any data.
Reproducibility	Given the logistical challenges of our experimental design we were limited to conducted the experiment once.
Randomization	Animals were first numbered from 1 to 297 and then randomly assigned to treatments.
Blinding	Blinding was not relevant to our study due to the nature of the experimental design and data collection. However, frogs from different treatments were were randomly distributed.

Did the study involve field work? ☒ Yes ☐ No

Field work, collection and transport

Field conditions	We collected the animals on a sunny day after a rainy day. The frogs started calling after 6pm, and the mean temperature of the collection night was 23°C.
Location	São Paulo, Brazil. Latitude: 23°38'02"S; Longitude: 46°40'54"

Access & import/export	To conduct our experiment, the following licenses were requested and approved: Unicamp Animal Care and Use Committee (CEUA #5398-1/2019), Instituto Chico Mendes de Conservação da Biodiversidade (SISBio #71780-1), and Sistema Nacional de Gestão do Genetic Heritage and Traditional Knowledge (SISGen #A8246D0).
Disturbance	This study caused no disturbance.

Reporting for specific materials, systems and methods

We require information from authors about some types of materials, experimental systems and methods used in many studies. Here, indicate whether each material, system or method listed is relevant to your study. If you are not sure if a list item applies to your research, read the appropriate section before selecting a response.

Materials & experimental systems

n/a	Involved in the study	
☒	☐	Antibodies
☒	☐	Eukaryotic cell lines
☒	☐	Palaeontology and archaeology
☒	☐	Animals and other organisms
☒	☐	Clinical data
☒	☐	Dual use research of concern

Methods

n/a	Involved in the study	
☒	☐	ChIP-seq
☒	☐	Flow cytometry
☒	☐	MRI-based neuroimaging
